# Ethics and contemporary urology practice: Setting out principles

**DOI:** 10.4103/0970-1591.56201

**Published:** 2009

**Authors:** A. Mohan

**Affiliations:** Department of Urology, St. John's Medical College & Hospital, Sarjapura Road, Bangalore 560 034, India

**Keywords:** Contemporary urology, ethics, principles

## Abstract

Several situations of great ethical implications are encountered by physicians in daily urological practice. Informed consent for interventions, selection of patients for operative demonstrations and educational workshops, enrollment of patients in clinical trials, and the use of technology are some issues that call for stringent application of ethical principles in decision making. The issues of autonomy, privacy, rights, duties, and privileges that arise have to pass the tests prescribed by contemporary social mores and regulations. Some of the issues encountered, principles applicable, and covenants and documents that guide decision making are discussed.

## INTRODUCTION

Issues of ethics gain plenty of importance in theoretical discussions in medicine. Several journals have published extensively on ethical debates covering various diverse aspects from teaching medicine to end-of-life issues. In the bustle of everyday clinical practice, some of these issues may not get the attention they merit. It would, therefore, be topical to discuss those that impact our routine clinical work. While a larger debate would certainly be both pertinent and beneficial, only a few common issues are taken up for discussion here.

## INFORMED CONSENT

Informed consent is the cornerstone of an evolved social system. Informed consent is routinely obtained from patients, or their legally empowered representatives, for the performance of any intervention that is likely to adversely impact their well-being. However, this is overwhelmingly viewed as a legal document, intended to protect the practitioner from allegations of malpractice and medical malfeasance. The ethics of this informed consent are as important as its legal validity.

An individual is vested with the responsibility to care for and control his body. Consequently, he is vested with the freedom to decide on matters that affect its integrity or well-being.[1] Any procedure that alters the internal or external body structure renders the body incapable of functioning to its full potential (e.g., due to pain, immobilisation, etc.) or takes away the individual's control over his body (e.g., anesthesia, sedation) require prior consent from the individual – even if the procedure is in the person's best interests.

Often overlooked are the ethical aspects of informed consent as it applies to children and sometimes the elderly. Obtaining consent from the parents (in the case of children) or the relatives (in the case of the infirm or elderly) is inadequate. Several societies and the International Convention on the Rights of the Child[2] recognise that children too are capable of understanding several aspects of their body and lives. Consent should therefore be obtained after the information has been made available to the child in a form that the child can understand – with all due care taken to answer questions and clarify doubts. It is expected that a pre-school age child is capable of understanding certain aspects of the body and life and can communicate preferences. A similar caveat would apply in the case of the infirm or elderly patients whose physical condition may be poor, but who possess the ability to understand, evaluate, and communicate choices. The process of consent should always involve them in a primary role. Notwithstanding the legal issues of age of consent and fitness to give consent, the ethics of consent mandate inclusion of the individual in the consent process. The real dilemma arises in the case of those with impaired sensorium, aphasia, or similar disability. Surrogate autonomy can be exercised by the family provided it is clear that the individual is not able exercise it. Caregivers (physicians included) should ascertain that the surrogates have the same degree of information available to them as would have been made available to the individual. Surrogate consent in the case of individuals under anesthesia or sedation is not to be encouraged except in extreme circumstances. This situation often arises when surgical procedures other than those for which the initial consent was given are required – either due to complications or unexpected findings. Wherever possible, it is necessary to wait for the patient himself to return to a state of sensorium that permits proper judgement and decision making. Blanket consent obtained to cover any eventuality is not ethical and certainly not legal.

### Demonstrations and Operative Workshops

Burgeoning educational initiatives pose ethical challenges that often escape attention. The underlying ethical contract between physician and patient undergoes a change when patients are screened for a workshop by one team, but are operated upon by another team of surgeons. Here, the patients' right to discuss the pros and cons with his care-provider is often subjugated – there is an imposed surrogacy. For example, when a patient is chosen to undergo a complicated surgical procedure by an expert, the process of explaining the surgery, the risks and befits, expected outcomes, possible complications, and the possible corrective measures should complications arise is executed by a surrogate team that by itself may not have the expertise in the procedure performed. This is compounded further by a near-complete absence of meaningful communications between the operating surgeon and the patient undergoing surgery.

A feasible solution would lie in the process of consent being undertaken by a suitable local expert who is conversant with the details. Alternatively, the operating surgeon should be available for the patient to discuss matters pertaining to his surgical procedure. Meaningful continuity of care after the workshop is necessary and should be ensured. Several concerns of professional ethics will remain about all this and more –the standard of care, sterility, protocols, and costs.

The dilution of indications for surgery is also a concern. The International Code of Medical Ethics[3] declares that *“A physician shall act only in the patient's interest when providing medical care which might have the effect of weakening the physical and mental condition of the patient”*. Choosing patients with marginal indications in order to demonstrate an announced or promised procedure is certainly not in the patient's best interests. It is also incumbent on the operating surgeon to ascertain that the procedure will indeed be in the patient's interest as laid down in the International Code of Medical Ethics. To take refuge behind the assertion that the local organisers will have taken care of all necessary documentation will take us back to the dark practices that necessitated the adoption of the Declaration of Helsinki.

Another aspect often overlooked is patient confidentiality. Revealing identities and announcing personal details are clear violations of patient's rights. This is very often the first casualty.

## CLINICAL TRIALS

The advent of drugs for treating various urological conditions and the need to obtain regulatory clearance for these drugs has brought clinical drug trials to the Urologists' doorstep. These trials are undoubtedly to be carried out in accordance with the Good Clinical Practice guidelines of the International Conference on Harmonisation of Technical Requirements for Registration of Pharmaceuticals for Human Use (ICH)[4] – together referred to as the principles of ICH – GCP. Patients in drug trials have even less access to information about benefits and risks than patients undergoing standard surgery. Often, even the physician is in the same boat.

The same rigour applied to surgical decision-making has to be applied here as well. The patient's right to information and autonomy in decision making should be protected. First of all, the physician-urologist has to apply his mind to the study protocol and understand the implications of the proposed treatment or intervention that is to be studied. In the case of drug trials, the data supplied by the sponsors should be carefully perused and independently analysed for consistency and reliability. The physician cannot shirk this responsibility and leave it for the patient to decide and then claim that it was the patient's decision. The offer for inclusion in the study should be made to the patient only if the physician has formed an informed, sound opinion himself that the intervention will benefit the patient. If after this the patient is unable to grasp the nuances of the medication, procedures involved, risks or benefits, it should be taken to mean that the patient has notconsented.

Relentless pursuit of patients for recruitment in trials is a practice driven by pressure from the sponsors to show performance. The physician's primary duty is to the patient, not the sponsor! It is here that the physician is at his most vulnerable. Ethics Committees rarely get to evaluate the financial agreements – there is sometimes a higher compensation per patient as the recruitment increases subtly pressurising the physician. Since the professional input that goes into the care of each patient is the same, the higher amount paid is clearly an inducement and no longer compensation, rendering it unethical.

The greatest ethical issue with clinical trials is the non availability of the data generated to the investigator – the patient-participant and the physician-participant both do not know the outcome of the trial. What benefit would then avail to the patient? How would the physician know if he acted in the patients' best interest or actually harmed the patient by recruiting them into the trial when proven alternative therapies were available? And then, there is the larger issue of non publication of negative outcomes – results that show that the treatment or intervention was of no use, or even harmful. International non industry agencies are slowly waking up to this.

## ETHICS OF TECHNOLOGY

The availability of advanced technology undoubtedly has made surgical treatment outcomes more successful over the years. This has also spawned several questions of ethics as follows:

- Has the patient been made aware of alternate treatments or technologies – for example, a physician who offers only Percutaneous nephrolithotomy without discussing Shock wave lithotripsy as a solution for the kidney stone.
- Has the patient been given enough information about the technology to be used and its limitations and risks?
- Is there enough evidence to support the safety of the new technology and/or its superiority over the earlier ones?
- Is technological experimentation being thrust on the patient in the name of new technology?
- Has the financial impact of the technology on the patient been evaluated and explained?
- Does the patient have access to continuity of care for complications directly attributable to the new technology? Are these reported?

Unlike the case of pharmaceutical trials, new technology in urology practice does not require approval from any Ethics Committee or Institutional Review Board. The Indian Council of Medical Research released its Ethical guidelines for Biomedical Research on Human Participants[5] in 2006, but these guidelines do not address the issue of technology, human rights, and patient choices.

## CONCLUSION

Ethics in clinical practice is an issue that has sometimes hovered only in the deep recesses of the physician's professional consciousness. This is no less true in the field of Urology. Issues of autonomy and rights have been codified for a few decades now, yet we find these applied erratically, if at all, in several spheres of urologic practice. The proper communication of benefits and risks should become an integral part of the ethical physician-patient relationship as this alone can help the patient take sound decisions. Calman[6] has identified three factors as being relevant in such communication: the certainty of the risk (the evidence base), the level of risk (how high or how low), and the effect(s) of the risk. This simple guide to communication should result in a more robust consent process. It is also a good guide to physicians who seek clarity on the issues involved in the communication of risks.

Hopefully, an understanding born out of diligent introspection and reference to established covenants and published documents will result in strengthening the ethical foundations of our current medical practice.
